# Validation of assay indicating method development of meloxicam in bulk and some of its tablet dosage forms by RP-HPLC

**DOI:** 10.1186/2193-1801-3-95

**Published:** 2014-02-18

**Authors:** Nalini Kanta Sahoo, Madhusmita Sahu, Podilapu Srinivasa Rao, N Sandhya Rani, JNV Indira Devi, Goutam Ghosh

**Affiliations:** Yalamarty Pharmacy College, Tarluwada, Visakhapatnam, 530052 AP India; School of Pharmaceutical Sciences, SOA University, BBSR, Kalinganagar, 751003 Orissa India

**Keywords:** RP-HPLC, Meloxicam, Quality control level

## Abstract

A novel, simple and economic reverse phase high performance liquid chromatography (RP-HPLC) method has been developed for the quantification of Meloxicam in bulk and tablet dosage form with greater precision and accuracy. Separation was achieved on Develosil ODS HG-5 RP C_18_, (15 cm × 4.6 mm i.d. 5 μm) column in isocratic mode with mobile phase consisting of acetonitrile: phosphate buffer(pH 3.4) (60:40) with a flow rate of 1 mL/min. The detection was carried out at 268 nm. The retention time of Meloxicam was found to be 2.09 min. The method was validated as per ICH guidelines. Linearity was established for Meloxicam in the range 20 – 120 μg/ml with R^2^ value 0.996. The percentage recovery of Meloxicam was found to be in the range 99.99-100.46%. The high recovery and low relative standard deviation confirm the suitability of the proposed method for the estimation of the drug in bulk and tablet dosage forms. Validation studies demonstrated that the proposed RP-HPLC method is simple, specific, rapid, reliable and reproducible for the determination of Meloxicam for Quality Control level.

## Introduction

**Figure 1:**
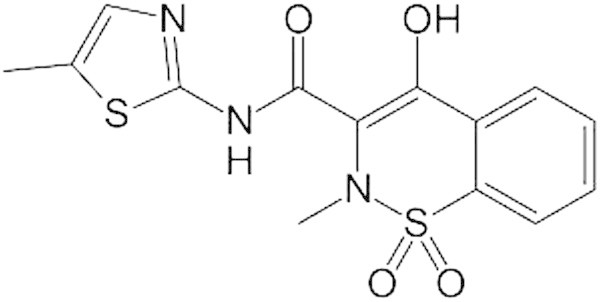
**Chemical structure of Meloxicam.**

Meloxicam (MEL), shown in Figure 1, is chemically, 4-hydroxy-2-methyl-N-(5-methyl-2-thiazolyl)-2H,1,2-benzothaizine-3-carboxamide-1, 1-dioxide. It is used for the treatment of osteoarthritis, rheumatoid arthritis, and pauciarticular and polyarticular course juvenile rheumatoid arthritis. Its analgesic, antipyretic and anti-inflammatory activity is due to the inhibition of COX-2 enzyme (Noble & Balfour 1996; Davies & Skjodt 1999). Meloxicam pharmacological activity is greatly affected by the substituents on parent oxicam moiety e.g. change in methyl to ethyl at 2 positions causes complete suppression of activity (Lemke & Williams 2008). Several analytical methods for the determination of meloxicam by flourimetry (Hassan 2002), capillary electrophoresis (Nemutlu & Kir 2003), spectrophotometry (Hassan 2002), HPLC (Arayne et al. 2005; Farzana & Pradeep 2009; Damle et al. 2009), LC/MS (Wiesner et al. 2003) and polarographic (Altıokka et al. 2000; Radi et al. 2001a; Altınöz et al. 2002; Radi et al. 2001b; Rao et al. 2005) have been reported. The aim of the present work was to develop and validate a sensitive RP-HPLC method that can be implemented for the quantification of meloxicam in bulk as well as in its tablet dosage forms.

## Experimental

### Materials

Pure Meloxicam (MEL) used as working standards, was purchased from Yarrow chem. Products, Mumbai, India. Tablets containing 15 mg and 7.5 mg of meloxicam (MUVERA15 and MOVAC) were obtained from Apollo Pharmaceuticals Pvt. Ltd, Visakhapatnam, India and used within their shelf life period. Acetonitrile and water (HPLC-grade) were purchased from Merck, India. All other chemicals and reagents employed were of analytical grade, and purchased from Merck, India.

### Instrumentation

The chromatographic system used comprised of Analytical technologies ltd UV 2230 UV–vis detector. Data integration was carried out using A-4000 version software. Samples were injected into Develosil ODS HG-5 RP C_18_ (5 μm, 15 cm × 4.6 mm i.d) column. An Analytical technologies ltd.sonicator was used for enhancing the dissolution of the compounds. A Wenster digital pH meter was used for pH adjustment.

### Chromatographic conditions

The high performance liquid chromatographic (HPLC) system used was operated isocratically with the column temperature maintained at 30°C, using a mobile phase composition of acetonitrile and phosphate buffer (pH adjusted to 3.4 with O-Phosphoric acid) in the ratio of 60:40 v/v at a flow rate of 1.0 mL/min within a run time of 7 min. Prior to use, the mobile phase was degassed by an ultrasonic bath and filtered by a Millipore vacuum filter system equipped with a 0.45 μm high vacuum filter. The drug was detected and quantified at 268 nm.

### Preparation of standard solutions

The stock solution was prepared by transferring 100 mg of Meloxicam into 100 mL volumetric flask. Then it was added with small amount of diluent [acetonitrile: water (50:50)], and the mixture was sonicated to dissolve and made up to volume with mobile phase. From this stock solution different concentrations were prepared to give final concentrations of 20 – 120 μg/mL for standard calibration curve.

### Assay of meloxicam from marketed tablets

Twenty tablets were accurately weighed and crushed to a fine powder in a mortar in each of the marketed formulation separately. An amount of the powder equivalent to 100 mg was transferred into a 100 mL volumetric flask and 10 mL of diluent was added to it followed by 10 ml of 0.1 N NaOH. The mixture was sonicated to dissolve the exipients and then made up to volume with mobile phase. Following 15 min of mechanical shaking, it was kept in an ultrasonic bath for 15 mins, and the solution was filtered through a 0.45 μm filter paper. Suitable aliquots (1 ml each) of the filtered solution were transferred to 50 ml volumetric flasks and made up to volume with mobile phase to yield six concentrations of Meloxicam (20 μg/mL). A 20 μL volume of the sample solution was injected into the chromatographic system, six times, under optimized chromatographic conditions. The peak areas were measured at 268 nm and concentrations in the samples were determined by interpolation from standard calibration curve of each drug previously obtained.

## Method validation

The method was validated in accordance with ICH guidelines (International Conference on Harmonization (ICH) of Technical Requirements for Registration of Pharmaceuticals for Human Use 2010). The parameters assessed were linearity, accuracy, and limit of detection (LOD), limit of quantification (LOQ), precision, reproducibility, robustness and system suitability.

### Accuracy

Accuracy was best determined by the standard addition method. Previously analyzed samples of Meloxicam API were added with standard drug solutions and are analyzed by the proposed method. Recovery (%), RSD (%) and bias (%) were calculated for each concentration.

Accuracy is reported as percentage bias, which is calculated from the expression

### Precision

Precision was determined as both repeatability and intermediate precision, in accordance with ICH guidelines. Repeatability of sample injection was determined as intraday variation and intermediate variation. For these determinations, single concentration (20 μg/ml) at different time intervals and different days, of the solution of Meloxicam API was used.

### Robustness

The concept of robustness of an analytical procedure has been defined by the ICH as “a measure of its capacity to remain unaffected by small but deliberate variations in method parameters”. To determine the robustness of the method experimental conditions are purposely altered and chromatographic characters are evaluated. Influence of small changes in chromatographic conditions such as change in flow rate (± 0.1 ml/min), wavelength of detection (±2 nm) and acetonitrile content in mobile phase (±2%) were studied to determine the robustness of the method.

### Limit of detection (LOD)

The limit of detection (LOD) of an analytical method may be defined as the concentration, which gives rise to an instrument signal that is significantly different from the blank. For spectroscopic techniques or other methods that rely upon a calibration curve for quantitative measurements, the IUPAC approach employs the standard deviation of the intercept (Sa), which may be related to LOD and the slope of the calibration curve, b, by

### Limit of quantitation (LOQ)

The LOQ is the concentration that can be quantitated reliably with a specified level of accuracy and precision. The LOQ represent the concentration of analyte that would yield a signal-to-noise ratio of 10.

Where, Sa is the standard deviation of the peak area ratio of analyte to IS (6 injections) of the drugs and b is slope of the corresponding calibration curve.

## Results and discussion

### Optimization of chromatographic conditions

**Figure 2:**
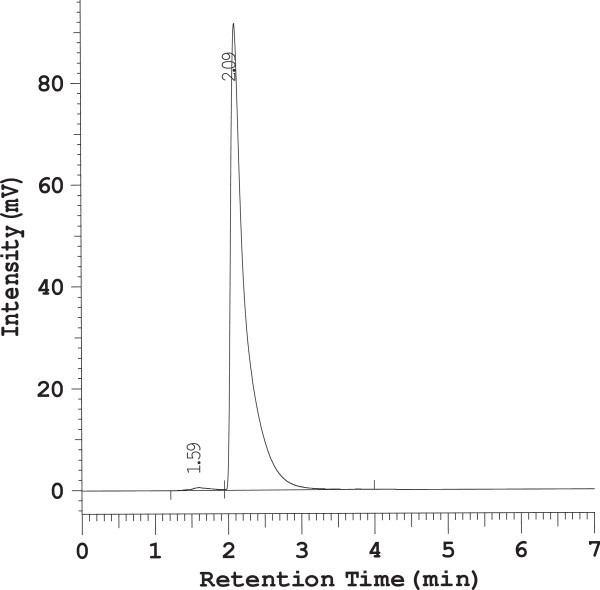
**RP-HPLC Chromatogram of Meloxicam standard drug.**

The chromatographic conditions were optimized by different means i.e. using different column, different mobile phase, different flow rate, different detection wavelength and different diluents for standard drug and marketed tablets are summarized in Table 1 and different chromatograms (Figure 2) are shown.

**Table 1:** **Results of optimization**

Column used	Mobile phase	Flow rate	Wave length	Observation	Result
Microbondapak C_18_, 5 μm, 50 × 4.6 mm i.d.	Methanol only	0.5 ml/min	254 nm	Low resolution below 1.	Method rejected
Phenomenex RP- C_18_, Luna 5 μm, 250 × 4.6 mm i.d.	Acetonitrile only	0.8 ml/min	358 nm	Resolution i.e. 1.2 but not satisfied	Method rejected
Microbondapak C_18_, 5 μm, 50 × 4.6 mm i.d.	Acetonitrile: phosphate buffer(pH7.8) = 70:30	1 ml/min	270 nm	Poor resolution i.e. 1.5	Method rejected
Phenomenex RP- C_18_, Luna 5 μm, 250 × 4.6 mm i.d.	Acetronitrile: phosphate buffer(pH 5.0 = 60:40	1 ml/min	270 nm	Poor resolution i.e. 1.2	Method rejected
Develosil ODS HG-5 RP C_18_, 5 μm, 15cm × 4.6 mm i.d.	Acetonitrile: phosphate buffer(pH 3.4) (60:40)	1 ml/min	268 nm	Sharp peak and good resolution i.e. 2.3	Method accepted

### Linearity & range

**Figure 3:**
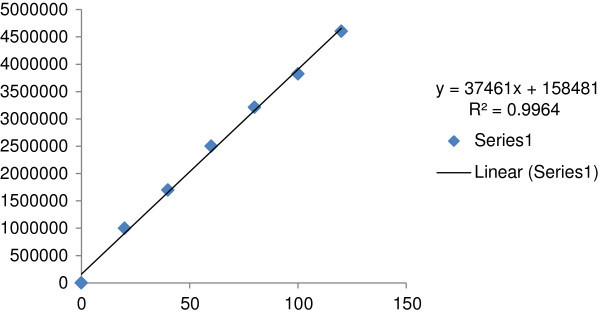
**Calibration curve for Meloxicam.**

The standard calibration curve(Figure 3) showed good linearity in the range of 20 – 120 μg/ml (Table 2), for Meloxicam API with correlation coefficient (r^2^) of 0.996. A typical calibration curve has the regression equation of y = 37461x + 158481 for Meloxicam.

**Table 2:** **Results of linearity and range**

Sl. no	Conc.	Area
1	20	1000024
2	40	1698687
3	60	2502000
4	80	3213014
5	100	3824253
6	120	4605216

### Accuracy: recovery study

The recovery of the method, determined by adding a previously analyzed test solution with additional drug standard solution at three levels of concentration, was 99.99 - 100.46%. The values of recovery (%), RSD (%) and % Bias listed in Table 3 indicate the method is accurate.

**Table 3:** **Results of recovery study**

Amount of drug added (μg) to analyte	Recovery from formulation			
	Mean amount (μg ) found (n = 6)	Mean % recovery	% RSD	% Bias
32	32.148	100.46	1.662	0.462
40	40.021	100.05	1.502	0.052
48	47.954	99.99	0.2757	−0.095

### Precision: Intra-assay & inter-assay

The intra & inter day variation of the method was carried out and the high values of mean assay and low values of standard deviation and % RSD (% RSD < 2%) within a day and day to day variations for Meloxicam revealed that the proposed method is precise (Table 4).

**Table 4:** **Results of intra-assay & inter-assay**

Conc. of Meloxicam (20 μg/ml)	Observed conc. of Meloxicam (μg/ml) by the proposed method				
	Intra-Day			Inter-Day	
	Mean (n = 6)	% RSD		Mean (n = 6)	% RSD
**9 A.M**	22.463	0.01	**Day 1**	21.89	0.08
**1 P.M**	22.463	0.01	**Day 2**	21.93	0.04
**5 P.M**	22.463	0.01	**Day 3**	20.99	0.01

### Robustness

Influence of small changes in chromatographic conditions such as change in flow rate (± 0.1 ml/min), Wavelength of detection (±2 nm) & acetonitrile content in mobile phase (±2%) studied to determine the robustness of the method are also in favor of (Table 5, % RSD < 2%) the developed RP-HPLC method for the analysis of Meloxicam API.

**Table 5:** **Results of robustness test**

Change in parameter	% RSD (n = 6)
Flow (0.9 ml/min)	0.14
Flow (1.1 ml/min)	0.02
Wavelength of Detection (270 nm)	0.09
Wavelength of detection (266 nm)	0.61
Acetonitrile: Phosphate buffer (62:38)	0.14
Acetonitrile: Phosphate buffer (58:42)	0.19

### LOD & LOQ

The Minimum concentration level at which the analyte can be reliable detected (LOD) & quantified (LOQ) were found to be 0.42 & 1.29 μg/ml respectively.

### Assay of meloxicam in tablet dosage forms

**Figure 4:**
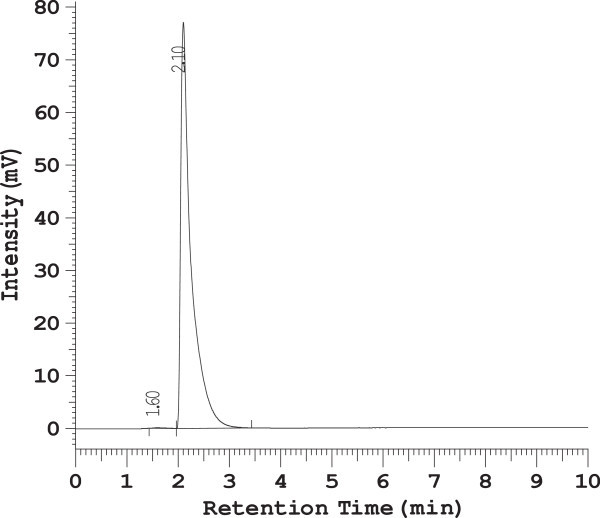
**RP-HPLC Chromatogram of Muvera 15 Tablet.**

**Figure 5:**
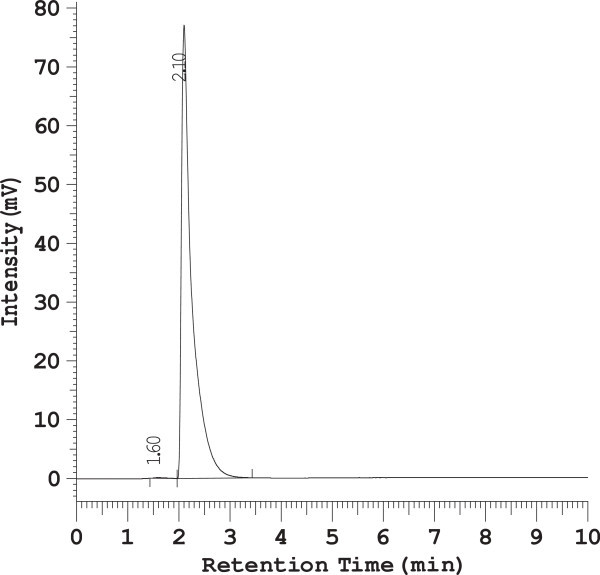
**RP-HPLC Chromatogram of Movac Tablet.**

Assay was performed by using the regression equation (y = 37461x + 158481, R^2^ = 0.996) obtained from the standard calibration curve of Meloxicam API. Results obtained are given in Table 6. The assay of Muvera 15 and Movac tablet containing Meloxicam was found to be 99.99 and 100.01% respectively as per the method. The chromatograms are represented in Figures 4 and 5.

**Table 6:** **Assay of meloxicam tablets**

S. No	Formulations	Standard peak area	Sample peak area	Labeled amount (mg)	Amount found (mg)	% Assay ± RSD
1	**Muvera 15**	1000024	1000011	15	14.99	99.99 ± 0.12
2	**Movac**	1000024	1000191	7.5	7.5	100.01 ± 0.02

The method is having linearity in the range 20 to 120 μg/ml with regression coefficient value 0.99. When compared to previous methods for recovery study, the % recovery is nearly 100%. Precision results are having low% RSD value i.e. below 0.1 in comparison to previous methods, which is an added advantage. Similarly in previous methods assay in marketed formulation is carried out by considering single formulation, but the present method uses two different formulations. Also at very low concentration it can be detected and quantified i.e. at 0.42 & 1.29 μg/ml, which is not observed in previous methods. For these reasons the method is proved to be novel in comparision to existed methods.

## Conclusion

A New RP-HPLC method indicating assay of MEL in bulk and in pharmaceutical dosage forms is established. This method is simple, reliable, linear, accurate, sensitive and reproducible as well as cost effective for the effective quantitative analysis of MEL in bulk and tablet formulations. The method was completely validated showing satisfactory data for all the method validation parameters tested and method is free from interference of the other active ingredients and additives used in the formulations. Therefore the method is suitable for use of the routine quality control analysis of Meloxicam in API or in pharmaceutical dosage forms.
